# Primary Tuberculosis of the Vallecula and Pyriform Sinus: A Case Report and Review of Literature

**Published:** 2015-03

**Authors:** Kamyar Iravani, Shahindokht Bassiri Jahromi, Mohammad Javad Ashraf

**Affiliations:** 1 *Department of Otorhinolaryngotogy. Shiraz University of Medial Sciences. Shiraz, Iran. *; 2 *Department of Mycology, Pasteur Institute of Iran, Tehran, Iran.*; 3 *Department of Pathology,Shiraz University of Medical Sciences, Shiraz,Iran.*

**Keywords:** Extrapulmonary, Pyriform sinus, Tuberculosis, Vallecula

## Abstract

**Introduction:**

Tuberculosis (TB) is a relatively prevalent infectious disease caused by a bacterium called mycobacterium tuberculosis. It primarily involves the lungs, but it can also affect other organs causing a variety of symptoms.

**Case Report:**

In this report, a rare case with primary involvement of pyriform sinus and vallecula due to tuberculosis in a 74-year-old woman who complained of odynophagia for 6 months, is reported. There were no clinical or radiological pulmonary findings.

**Conclusion:**

The authors point out the epidemiological importance of tuberculosis and the need for more suspicion when dealing with uncommon lesions to make an early diagnosis.

## Introduction

Tuberculosis (TB) is a disease, which is prevalent all over the world, especially in Asian and African countries. Annually, according to WHO, two million of these patients die. In recent years, in conjunction with an increase in positive HIV cases, the prevalence rate in these patients has increased (1).

This disease is caused by bacillus mycobacterium tuberculosis and primarily affects the lungs. 

Lung disease symptoms include cough, bloody sputum, and chest pain. Almost all organs and tissues may be affected to varying degrees by the disease with or without pulmonary infection (2).

Extrapulmonary tuberculosis is more frequently seen in patients who are in an immunodeficiency state, such as HIV infection. Common organs affected by extra-pulmonary TB include the pleura, meninges, central nervous system, lymphatic system, genitourinary organs, bones, and joints, and each involves its own symptoms. Involvement in the head and neck is common and includes the lymph nodes, salivary glands, larynx, nasopharynx, and ears (2,3). In this report, a rare case of primary tuberculosis of vallecula and pyriform sinus in an otherwise healthy woman, who also showed dysphagia caused by a mimicking mass, is reported.

## Case Report

A 74-year-old woman showed a chronic and progressive sore throat and dysphagia lasting 6 months. The patient had difficulty especially swallowing solids. There was no history of smoking, alcohol consumption, cough, or any respiratory problem.Past and family history was not significant. The patient had no fever, no night sweating, nor any loss of appetite. She had been on antibiotic treatment with the impression of bacterial infection, but did not respond to them.

During general physical examination, the patient was observed to be thin and did not show cervical lymphadenopathy. During oral examination, the tonsils did not show enlargement and congestion. Examination of the chest was within normal limits. During indirect laryngoscopy, the larynx was normal but a pathology was seen in the left pyriform sinus. The pharynx showed a large 4 cm ulcerative and granular lesion on the posterolateral hypopharyngeal wall.

**Fig 1:**
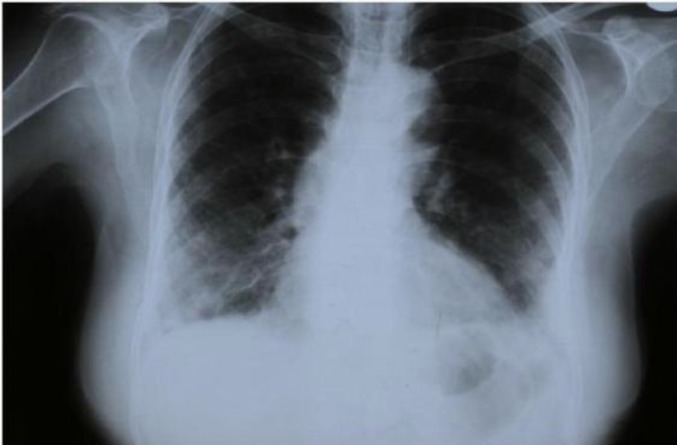
Chest x-ray (CxR) Showing not any pathologic sign

Routine tests revealed Hb-12.8 g% and ESR-7 mm. Liver and renal function tests were normal. X-ray of the chest was within normal limits (Fig. 1).

**Fig 2:**
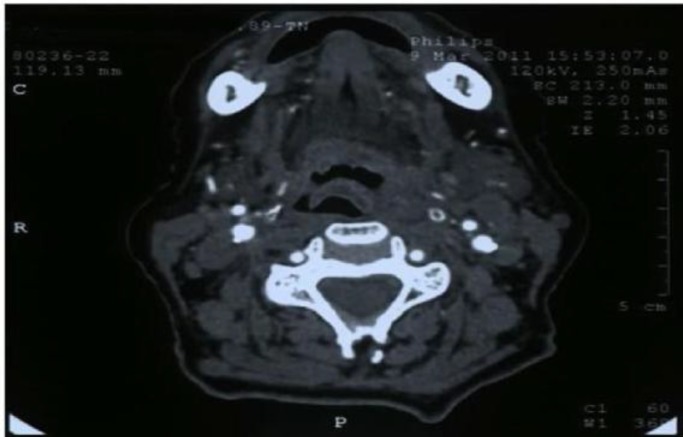
Axial computed tomography (CT) scan of neck showing a left pyriform sinus mass

**Fig 3:**
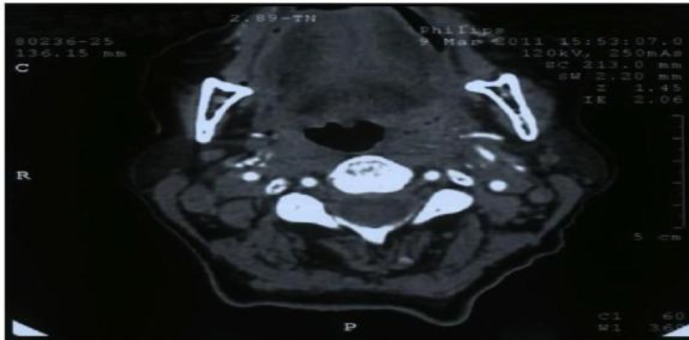
Axial computed tomography (CT) scan of neck showing an ill defined mass of hyp

The patient was HIV seronegative. Chest sputum test was negative. CT of the neck showed an ill-defined mass about 3×2cm in the left hypopharynx and posterior pharyngeal wall near the supraglottic area involving the pyriform sinus (Figs.2,3).

**Fig 4:**
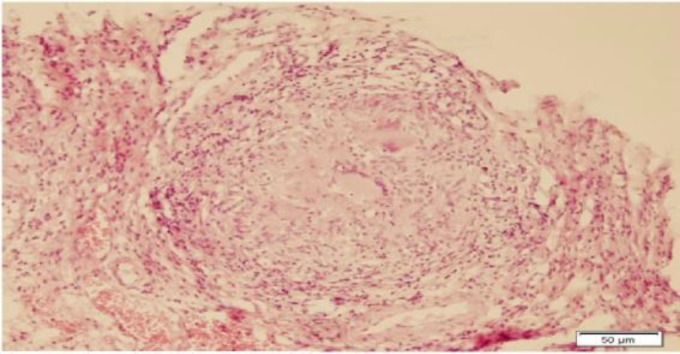
Noncaseating granuloma with giant cells and epithelioid histiocytes, H&E stain (x100)

**Fig 5:**
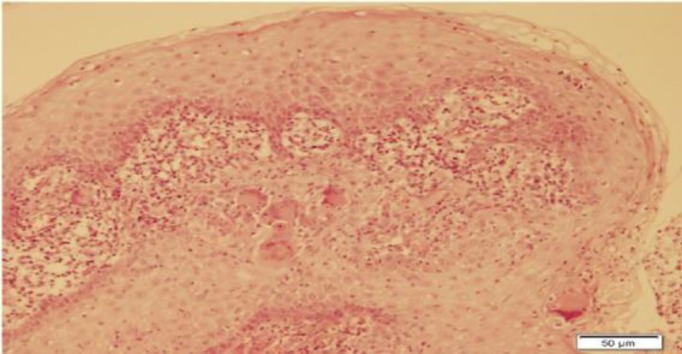
Ginat cell and epithelioid histiocytes under the squamous epithelium of pharynx, H&E stain (x100

**Fig 6:**
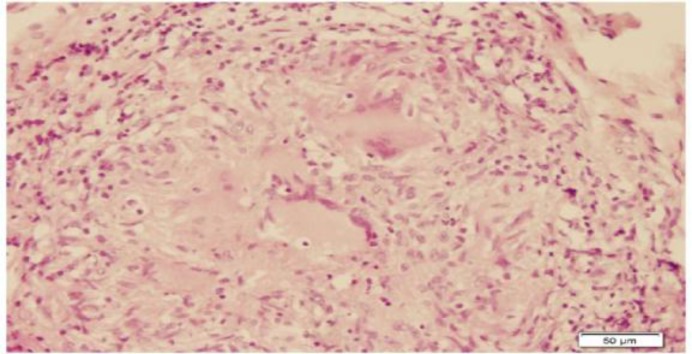
High power view of the granul omatous inflammation H&E stain (x200)

The patient underwent direct laryngoscopy and a biopsy was performed. Histopatho- logical examination of the mass revealed noncaseating granulomatous inflammation with Langhans giant cells (Fig. 4-6).

**Fig 7:**
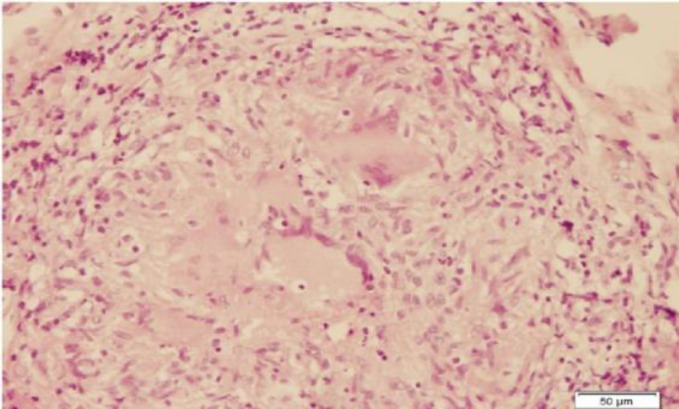
Acid stain of granuloma which was negative, ZN stain(x200

Ziehl-Neelsen stain for acid-fast bacillus was negative (Fig.7).

**Fig 8:**
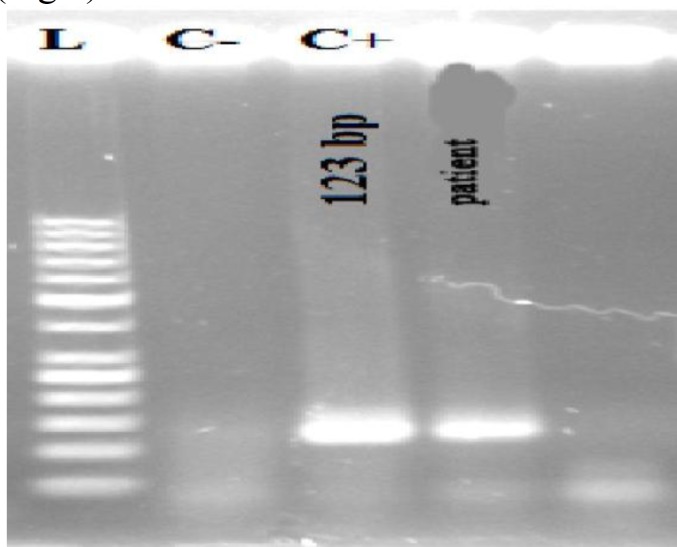
Agarose gell electrophoresis of mycobacterium tuber culosis of PCR reaction of the patient shows positivity compared to control

So PCR for mycobacterium was done on tissue paraffin block that was positive (Fig.8).

Tuberculin test showed induration of about 22mm. After the confirmation of the diagnosis, treatment commenced with isoniazid, rifampicin, The treatment consisted of pyrazinamide and ethambutol administration for a period of 2 months, followed by rifampicin and isoniazid administration for 6 months. The patient responded to anti-tuberculosis treatment dramatically and the pathology disappeared in the next laryngoscopy performed four weeks later. During the follow up, the patient showed complete regression of the endo-laryngeal lesion.

## Discussion

Primary tuberculosis of the hypopharynx is a very rare case of tuberculosis. Pharyneal and hypopharyngeal tuberculosis is more prevalent in the age range of thirty to sixty years. The tonsils are the most common organs involved. The most common symptoms are difficulty swallowing and odynophagia (4). Often these infections do not show primary pulmonary involvement and bacteria seems to spread hemato- genously or via the lymphatic system (5,6).

AI-Serhani reported one case of hypopharyngeal tuberculosis with the initial symptom of dysphagia, which is similar to the patient presented in this case report (7).

SaLC et al. reported a case of primary pharyngolaryngeal tuberculosis in a pregnant woman with a competent immune system. The chief complaint of this patient was dysphagia (4).

Tsai et al. reported a case of left pyriform sinus fistula due to tuberculosis that presented itself in the form of a neck mass with abscess formation and thyroid gland inflammation (8).

Goyal A. et al. also reported a rare case of pyriform sinus tuberculosis, which was grossly similar to a malignant hypopharyngeal mass (9). There are a wide range of differential diagnoses that causes difficulty in the diagnosis of tuberculosis. The most important differential diagnosis in these patients is carcinoma, which is grossly very similar and has clinical manifestations similar to tuberculosis. Other differential diagnoses include lymphoma, minor salivary gland tumors, neurogenic tumors, and Wegener's disease. Diagnosis is based on the isolation of mycobacterium tuberculosis bacillus and histopathological confirmation (4,6,10,11). 

Treatment includes isoniazid with a combination of two or three of rifampin, ethambutol, and pyrazinamide drugs for a duration of six to twelve months. Pyrazi- namide and ethambutol are recommended for the first two months of treatment. After two weeks of treatment, symptoms improve and contagiousity of the micro-organism is significantly decreased (1,2,11).

**Extrapulmonary tuberculosis requires a longer duration of treatment. At least, twelve months of drug treatment is recommended to control the infection**.

## Conclusion

Isolated and primary tuberculosis of the pyriform sinus in the absence of pulmonary tuberculosis is a rare entity. Otolaryngo- logists should consider hypo-pharyngeal tuberculosis as one of the differential diagnoses in lesions especially in the countries where tuberculosis is endemic or in HIV positive patients after ruling out malignant lesions.
